# Characterization of the gut microbiome in wild rocky mountainsnails (*Oreohelix strigosa*)

**DOI:** 10.1186/s42523-021-00111-6

**Published:** 2021-07-17

**Authors:** Bridget Chalifour, Jingchun Li

**Affiliations:** 1 Department of Ecology and Evolutionary Biology, University of Colorado Boulder, 1900 Pleasant Street, 334 UCB, Boulder, CO 80309 USA; 2 Museum of Natural History, University of Colorado Boulder, 265 UCB, Boulder, CO 80309 USA

**Keywords:** Microbiome, Mollusk, Gastropod, Gut, 16S rRNA gene, Next-generation sequencing

## Abstract

**Background:**

The Rocky Mountainsnail (*Oreohelix strigosa*) is a terrestrial gastropod of ecological importance in the Rocky Mountains of western United States and Canada. Across the animal kingdom, including in gastropods, gut microbiomes have profound effects on the health of the host. Current knowledge regarding snail gut microbiomes, particularly throughout various life history stages, is limited. Understanding snail gut microbiome composition and dynamics can provide an initial step toward better conservation and management of this species.

**Results:**

In this study, we employed 16S rRNA gene amplicon sequencing to examine gut bacteria communities in wild-caught *O. strigosa* populations from the Front Range of Colorado. These included three treatment groups: (1) adult and (2) fetal snails, as well as (3) sub-populations of adult snails that were starved prior to ethanol fixation. Overall, *O. strigosa* harbors a high diversity of bacteria. We sequenced the V4 region of the 16S rRNA gene on an Illumina MiSeq and obtained 2,714,330 total reads. We identified a total of 7056 unique operational taxonomic units (OTUs) belonging to 36 phyla. The core gut microbiome of four unique OTUs accounts for roughly half of all sequencing reads returned and may aid the snails’ digestive processes. Significant differences in microbial composition, as well as richness, evenness, and Shannon Indices were found across the three treatment groups.

**Conclusions:**

Comparisons of gut microbiomes in *O. strigosa* adult, fetal, and starved samples provide evidence that the host internal environments influence bacterial community compositions, and that bacteria may be transmitted vertically from parent to offspring. This work provides the first comprehensive report on the structure and membership of bacterial populations in the gastropod family Oreohelicidae and reveals similarities and differences across varying life history metrics. Strong differentiation between these life history metrics demonstrates the need for wider sampling for studies of dynamics of the snail gut microbiome.

**Supplementary Information:**

The online version contains supplementary material available at 10.1186/s42523-021-00111-6.

## Introduction

Complex microbial communities living in animal gastrointestinal tracts are known to play critical roles in a variety of biological processes [1]. These communities can be comprised of a mix of horizontally acquired exogenous microbial taxa, and “core” taxa that confer selective advantages to the host and may be acquired vertically through the parent or horizontally [2]. Symbiotic gut bacteria can provide many beneficial services that affect host fitness, including nutrient absorption, digestive capabilities, immune response, and adaptation to abiotic challenges [3–7]. Maintaining a balanced microbial community and preventing dysbiosis (negative health effects due to an imbalanced microbiome) is therefore crucial to the host’s health. Varying factors can influence animal gut microbiome compositions, including host diet, pathogens, seasonality, and disease [8–12]. Gut microbiomes may also shift based on host developmental stages [12–17].

Gut microbiomes have been characterized across the animal kingdom, but with a heavy focus on humans and commercially or medically important vertebrate species [18–20]. Invertebrates make up more than 97% of animal species alive on Earth, but only a minority of microbiome studies focus on invertebrate subjects [21]. Also, many large-scale studies of non-human gut microbiomes often use captive individuals, and the results may not necessarily generalize well to natural populations. We severely lack understanding of invertebrate gut microbiomes, especially those of wild, undomesticated species.

Gastropods are one of the most diverse invertebrate groups. Terrestrial snails, in particular, are ecologically important as herbivores, detritovores, and prey species. Terrestrial snail populations have been declining alarmingly at the global scale due to increased human induced pressures [22]. Few studies have analyzed snail microbiomes [23]. There is an urgent need to understand how snails and their associated microbiomes are being impacted or adapt to the changing environment, as this information has become increasingly important for conservation biologists and land managers [24]. One of the initial steps is to start characterizing microbiomes of representative snail species.

**Fig. 1:**
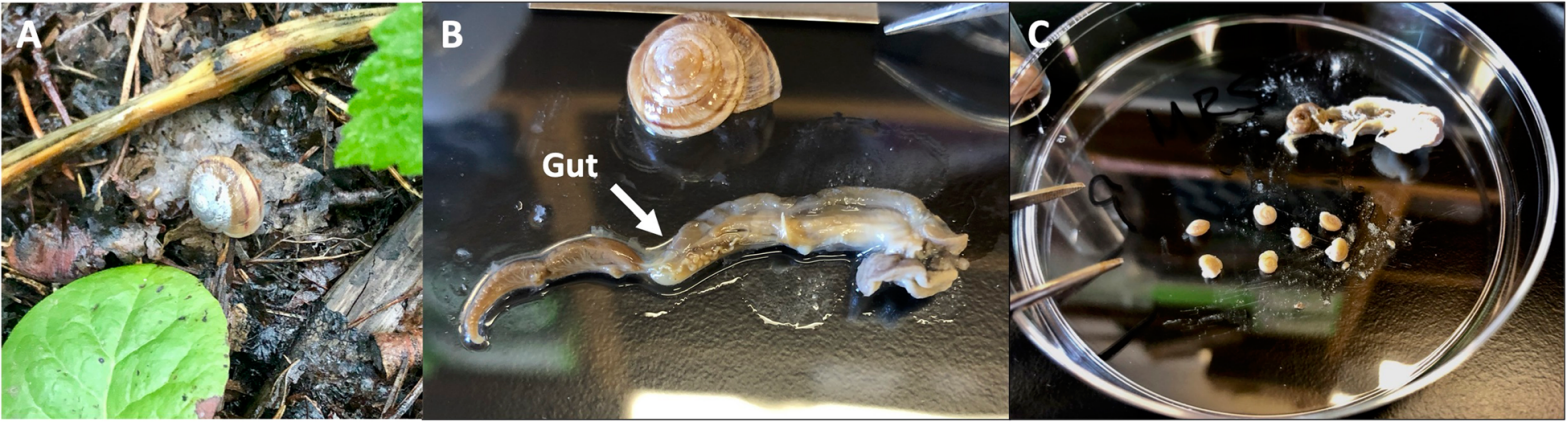
**A** An adult *Oreohelix strigosa* in its native habitat*,* **B** a dissected and complete *O. strigosa* internal body showing gut location, alongside shell, **C** extracted fetal snails dissected from adult *O. strigosa*

The genus *Oreohelix* is the most diverse group of land snails in North America, encompassing 79 species [25, 26], and contain the dominant malacological fauna of the Rocky Mountains [26, 27]. *Oreohelix* snails serve important ecological roles as detritivores and herbivores, feeding on both decaying wood and herbaceous vegetation [28]. They are an important source of calcium to other species, such as avian predators which use calcium for egg development [29, 30]. *Oreohelix* are found in a multitude of habitats, varying from grassy fields to the talus slopes of the Rocky Mountains [31]. Unlike many other pulmonate snails, *Oreohelix* species including *O. strigosa* are ovoviviparous, meaning embryos are brooded internally until the parent gives birth to live young [32, 33]. Ovoviviparity has been suggested to be advantageous in land snails because it allows parents to retain their young to a later stage of development, and to release them when environmental conditions are favorable (e.g., at the beginning of the growing season) [33, 34]. Embryo retention could give young further advantages, as it may facilitate vertical transmission of a microbiome from parent to offspring. Ovoviviparity in *O. strigosa* provides a valuable resource for comparing adult microbiomes to those of unborn offspring. Over half of the family Oreohelicidae is listed as critically imperiled or imperiled by NatureServe [35, 36]. *O. strigosa’s* ovoviviparity reduces the number of young that can be produced by a mother, compared with egg-laying snails [33]. This can make population recovery after perturbations very difficult. Because of their low vagility and vulnerability to desiccation, many populations exist only within certain geographically isolated moist microhabitats in rocky outcrops or canyons. As such, *O. strigosa* populations can have an island-like distribution, with many existing in “sky island” habitats of mountainous regions surrounded by low-lying areas that restrict any dispersal [37]. Sky island habitats can foster genetically distinct populations, and their restricted distribution makes species existing within these areas important to study for their conservation [37]. Within the genus *Oreohelix,* some species (e.g., Black Hills Mountainsnail [*O. cooperi*]) are already being listed as ‘threatened’ at the state level. Others, such as the Rocky Mountainsnail (*Oreohelix strigosa*, Fig. 1), are generally secure, but reported to be declining. *O. strigosa* is distributed from Southern Montana to Northern Arizona, and from Eastern Washington to Eastern Colorado [38]. This species was once common and widespread across the Colorado Front Range in the early 1900s, since then, observers have found that fewer populations remain ([32, 39], personal observation). The Rocky Mountainsnail may be dwindling, despite being listed as abundant and non-threatened (G5 status, secure) [35].

To our knowledge, the gut microbiome of *Oreohelix strigosa* has never been systematically characterized, despite its ecological importance. Valuable baseline data can be derived from characterizing host-associated microbial communities [40]. These data will lead to better detection of snail dysbiosis, which may be caused by any number of environmental stresses [40]. In this study, we used 16S rRNA gene amplicon sequencing to investigate the gut microbial communities of three populations of *Oreohelix strigosa*. We characterized core and unique gut microbes by comparing developmental stages of the snails, and by starving a sub-group of the snails. This study contributes to our understanding of the basic characteristics of the gut microbiome in Rocky Mountainsnails, and thereby promotes ex situ conservation should these snails become threatened.

## Materials and methods

### Specimen sampling and preservation

Between July and September of 2018 when terrestrial snails from the Rocky Mountains are most active, we collected living samples of *Oreohelix strigosa* from three geographically close locations within the Colorado Front Range: (1) the University of Colorado Mountain Research Station in Ward, Colorado, USA, hereafter: MRS; (2) Frisco, Colorado; and (3) Vail, Colorado (Table 1). These freshly collected samples (*N* = 72, including fetal snails found within adults) included 29 from the MRS, 8 from Frisco, and 35 from Vail. We used a qualitative collection method (i.e., collections made by direct visual searching) to collect specimens for this study, as snails present on tree trunks and limbs or vertical rock ledges are usually not effectively collected with quadrat samples. *O. strigosa* are most likely to be found under the bark of trees (specifically preferential to aspens), near bases of rocks, under logs, and in other microhabitats that might be missed in a completely random selection of samples. Therefore, the qualitative collection approach can maximize collection success [41]. All collections were taken with the appropriate permitting for invertebrates.

**Table 1:** Breakdown of sampled snails and their associated locations and treatment groups

Location:	GPS coordinates:	Adults, non-starved:	Fetal:	Adults, starved:	Total:
Mountain Research Station	40.0314420, − 105.5394289	14	12	1	27
Vail	39.6449151, −106.3146577	30	0	3	33
Frisco	39.5752723, −106.1181343	6	0	2	8

Live snails were drowned in distilled water and preserved in 95% ethanol for 24 h, then transferred to and kept in 80% ethanol for permanent preservation as they were extracted. This methodology has worked best on *O. strigosa* for follow-up research and dissection, allowing for increased plasticity in the tissue and easy removal of the whole body from the shell for more precise gut dissections.

To minimize the occurrence of transient bacteria within the gut, directly after collection a small subset of snails of each locality were kept under starvation conditions with a natural photoperiod for approximately one week. They were given no food supplement of any kind. These starved snails were then sacrificed in the same manner as the other live snails.

*O. strigosa* is an ovoviviparous species which gives live birth to its offspring, unlike some other land snails which lay eggs. Unborn fetal snails were dissected directly from the bodies of adult, already sacrificed snails. Here, we refer to individuals with unborn fetal snails inside of them as gestating. As there was no way to determine which snails were gestating ahead of time, the fetal snails came from three non-starved adult snails from the same location (MRS), who happened to be gestating at the time of collection. Fetal snails were dissected from the parent, and their shells were washed with 70% ethanol to remove external microbial communities. Due to the low amount of biomass in each fetal snail, the entire body was used as tissue sampling for DNA extraction, using the same kit and methodology.

In total, we used 50 non-starved adult snails, 6 starved adult snails, and 12 fetal snails.

### DNA extraction, PCR amplification, and sequencing

All dissections were performed aseptically, using sterile instruments. The soft body of the adult snail (Fig. 1) was removed by using fine forceps to gently pull the body out in its entirety by the foot. The digestive tract was carefully isolated from the body, as identified by its unique color and tissue texture, then, a portion of gut was collected from the stomach to the anus (excluding the anus). We then re-preserved all shell and body parts individually in 80% ethanol after the necessary tissue was removed. Information regarding museum catalog numbers and supplementary metadata can be found in Additional file 1.

We extracted genomic DNA from the snail gut tissue using the E.Z.N.A. Mollusk DNA Extraction kit (Omega Bio-tek, Norcross, GA) according to the manufacturer’s instructions. This kit was chosen because it resulted in the highest DNA yield among two other tested protocols we have assessed: (1) the Qiagen Powersoil kit, and (2) the Qiagen kit with an additional preliminary tissue drilling step. A positive control of *E. coli* was included to check protocol success up through gel electrophoresis, as well as negative controls of extraction and PCR blanks. The V4 hypervariable region of the 16S rRNA gene was amplified by PCR using the 515F/806R primer pair modified to include Illumina adapters and appropriate error-correcting barcodes, as comparable with other microbiome studies [42–47]. PCR amplification protocol was taken from the Earth Microbiome Project protocol for 515F/806R [47]. Library preparation and sequencing was facilitated by the Center for Microbial Exploration at the University of Colorado Boulder. 150 bp single indexed paired-end reads were generated on an Illumina Miseq platform PE300 (Illumina Corporation, San Diego, CA, USA) using a 2-by-150-bp paired end chemistry with the MiSeq V2 300-cycle kit (Illumina, San Diego, CA, USA) at the University of Colorado Next-Generation Sequencing Facility through BioFrontiers (Boulder, CO, USA). Samples were sequenced on one Illumina MiSeq run.

Data were processed using the USEARCH10 pipeline [48]. Reads were merged with a minimum overlap of 16 bp (usearch8 -fastq_mergepairs). Trimmed reads were quality filtered with a max error rate of 1.0 (usearch10 -fastq_filter; 96.2% passed). Unique sequences were identified using usearch10 -fastx_uniques which clustered as 99% 16S rRNA operational taxonomic units (OTUs) with usearch10 -cluster_otus uniques.fa. OTUs were classified taxonomically using the GreenGenes 13_8 database [49]. We removed those OTUs that were classified as mitochondria or chloroplasts, and we ignored any samples that yielded fewer than 2000 reads per sample. Four of the 72 snail gut samples (all adult, non-starved samples, one from the MRS, three from Vail) failed to meet this threshold for sequencing depth and was excluded from downstream analyses.

Species identification was confirmed using the COI mitochondrial gene amplified using primer sets LCOI490 5′-GGTCAACAAATCATAAAGATATTGG-3′ and HCO2198 5′-TAAACTTCAGGGTGACCAAAAAATC-3′ to compare against the most up to date *Oreohelix* family tree COI sequences from Linscott et al. [36].

### Statistical analyses

Statistical analyses were conducted in R version 4.0.2 [50] using the {MCToolsR} package [51]. We sought to analyze the richness, evenness, and alpha- and beta- diversity across all samples to understand the gut microbiome broadly at the snail species level, and between treatment groups of adult non-starved snails (*n* = 50), adult starved snails (*n* = 6), and fetal snails (*n* = 12). To include our samples while dropping out extraction blanks, we chose a sequencing depth of 2000 reads for rarefaction. Rarefied samples were used for downstream analysis. For all statistical analyses in the present study, a *p*-value < 0.05 was considered significant.

We tested if the taxonomical bacterial compositions among snails were different. Non-metric multi-dimensional dimensional scaling (NMDS) plots were used to visualize the similarities of gut microbial communities between the different snail groups. To test for significant differences in gut microbiome composition among treatment groups, we ran a permutational analysis of variance (PERMANOVA) using the adonis function in the R package, {vegan} [52] using Bray-Curtis dissimilarity. We re-ran a PERMANOVA using only samples found in one location (MRS) with all three treatment groups present, to confirm whether age and starvation are significant factors when location differences were eliminated.

We characterized microbiome compositions across the three snail groups using several indicators, including species richness, evenness, and Shannon diversity index. Species richness, evenness, and Shannon diversity index for each individual were calculated using the {vegan} package [52]. Then, we statistically tested if these diversity indices were different from one another. As measures of richness, evenness, and Shannon index were not normally distributed, as shown by plotting histograms and running Shapiro-Wilks tests, we used the non-parametric alternative of the Kruskal-Wallis test to determine significant differences between groups in lieu of an ANOVA. The Dunn test was conducted as a post-hoc test in R using the “dunnTest” function of the {FSA} package [53]. Displaying results of the Dunn test in compact letter form was done using the {rcompanion} package [54]. *P*-values were adjusted for multiple comparisons using the Bonferroni correction using the “p.adjust” function in {stats} [50]. All values shown in boxplots represent the standard error of the mean.

As there were uneven sample sizes across the three treatment groups, we used boot-strapping analysis to determine if significant differences between groups persisted when sample sizes were equal. To do this, the 6 starved snails and 12 fetal snails were compared to random samples of 6 and 12 adults respectively in each bootstrapped replicate. This sampling procedure was repeated 100 times, and for each replication the Kruskal-Wallis test was conducted on the balanced sample.

Venn analysis was done using the {VennDiagram} package [55]. Graphs were created using {ggplot2} [56] and {paRkpal} [57] packages.

## Results and discussion

To our knowledge, this is the first study to characterize the gut microbiome, or any microbiome, of a member of the land snail family Oreohelicidae. In these results, we will present the overall taxonomic composition of *O. strigosa* gut microbiomes and members of the core microbiome. Then, we present differences between the three treatment groups of adult, fetal, and starved snails both taxonomically, and in terms of diversity metrics.

### Overall species microbiome

#### Sequencing statistics

Next-generation sequencing of 16S rRNA sequences was employed to assess the gut microbiomes of 50 non-starved adult snails, 6 starved adult snails, and 12 fetal snails. We obtained 2,714,330 reads in total from the 68 samples. Of those reads, 7056 unique OTUs were identified. The number of OTUs per sample ranged from 23 to 1559. The number of reads per OTU ranged from 0 to 39,012 reads (this was an OTU from genus *Mycoplasma*), and the average number of reads per OTU was 384.68. The alpha- diversity indices indicate a high diversity of the *O. strigosa* bacterial community, as compared with other snail species. For example, there were 7065 total OTUs in *O. strigosa*, versus 1196 OTUs in freshwater snail *Radix auricularia* [58]. The invasive land snail *Lissachatina fulica* only had 228 OTUs [8].

These discrepancies may be driven by the snails’ differential dietary and habitat needs. *O. strigosa* primarily feeds on decaying lignocellulosic matter, rather than fresh vegetation [28]. Lignocellulosic matter may be more difficult to digest, thus calling for additional commensal bacteria to assist digestion. Alternatively, in termites, a subset of microbes appears to be responsible for digesting lignocellulosic matter [59]. However, microbial richness decreased only when termites were fed a singular, specific diet of straw, supporting the idea that microbial diversity declines when the diet solely consists of lignocellulosic matter [59]. *Oreohelix strigosa* may therefore have a much more diverse diet than expected and need additional gut microbe symbionts. *Radix auricularia* is a freshwater species that likely has different dietary needs than a terrestrial snail, consuming more aquatic plants and algae than woody matter and dry leaf litter. Dietary diversity may also be directly correlated with habitat diversity and may in turn impact bacterial diversity in guts. The land snail *Lissachatina fulica* does have a wide-ranging diet, as it is an invasive species that adapts to a broader range of environments. In Cardoso et al. [8], *Lissachatina fulica* was collected from one locality in its invasive range. This narrow sampling range might be driving the lower number of OTUs recovered; it is also worthy to note that methods of OTU classification, including similarity thresholds and sequencing technologies, vary between these studies. Further broader sampling may reveal different microbiome diversity patterns.

#### Taxonomic composition of gut bacteria

**Fig. 2:**
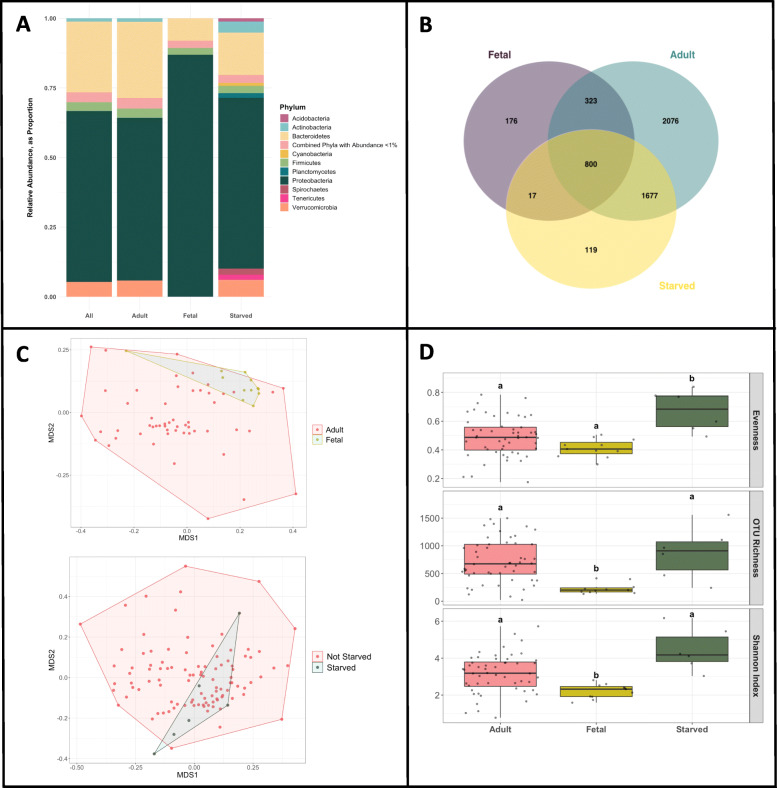
**A** Relative abundance of bacterial phyla contributing at least 1% in each snail sample set. **B** Three-way Venn diagram of the microbial OTU composition in the guts of members of *O. strigosa* from adult, fetal, and starved groups. **C** Non-metric multi-dimensional dimensional scaling analyses for (upper) adult vs. fetal samples (PERMANOVA: *p*-value < 0.001, *R*^*2*^-value = 0.095) and (lower) starved vs. non-starved samples (PERMANOVA: *p*-value < 0.002, *R*^*2*^-value = 0.038). **D** Diversity boxplots showing differences in species richness, species evenness, and Shannon Index of adult, fetal, and starved groups. Jitter shows distribution of samples. Letters above bars indicate significant differences

The taxonomic composition of *Oreohelix strigosa* gut microbiomes proved to be highly diverse (Fig. 2A). A total of five phyla accounted for 96.48% of the total sequences across all samples. Proteobacteria (61.29%) and Bacteroidetes (25.44%) were the most dominant bacterial phyla followed by Verrucomicrobia (5.36%), Firmicutes (3.22%), and Actinobacteria (1.17%). A total of 31 other classified phyla each comprised less than 1% of the relative abundance; and one phylum was not classified. Proteobacteria contained the largest number of OTUs (2835) which belonged to the following classes, in order from greatest contribution to least: alpha-, gamma-, beta-, delta-, epsilon-, and zeta- proteobacteria, with alphaproteobacterial contributing the majority of OTUs (1613), followed by Bacteroidetes with 1146 OTUs and Actinobacteria with 956 OTUs.

Our results showed that within the phylum Proteobacteria, gammaproteobacteria constitutes the largest proportion, and within gammaproteobacteria, the order Enterobacteriales is the most abundant. Members of Enterobacteriales are widely common gut bacteria, having been reported across animal phyla including vertebrates and invertebrates [60].

There were 20 identifiable bacterial families with > 1% abundance across all samples, which accounted for 82.03% of the total sequences. Among them, Enterobacteriaceae (41.95% of sequences), Sphingobacteriaceae (15.84%), Flavobacteriaceae (4.75%), Pseudomonadaceae (4.48%), and Verrucomicrobiaceae (4.43%) were the most common families.

Over half (53.15%) of genera were not classified. There were 10 genera with > 1% abundance across all samples; these included *Sphingobacterium*, *Pseudomonas*, *Flavobacterium*, *Serratia, Pedobacter, Acinetobacter, Sphingomonas, Yersinia, Enterococcus,* and *Luteolibacter*, with abundances ranging from 1.14 to 13.06%.

The proportional dominance of members of these taxonomic levels is consistent with other snail gut microbiome studies. The phyla Proteobacteria, Bacteroidetes, and Actinobacteria were also the dominant phyla in Planorbid snail intestines [61]. Proteobacteria (and within, specifically alpha- and gammaproteobacterial classes) similarly has been identified as the dominant bacterial phylum in the gut microbiome in both distantly related snails, and snails from freshwater and terrestrial environments, including *Biomphalaria pfeifferi*, *Bulinus africanus*, *Helisoma duryi* [61], *Lissachatina fulica* [8, 9], *Helix pomatia* [11], and *Radix auricularia* [58]. However, our results show a striking higher relative abundance of Proteobacteria (61.29%) compared to that of other snails. For example, in the big-ear radix, *Radix auricularia*, Proteobacteria only accounts for 36.0% of sequences in juvenile snails and 31.6% in adults [58]. Other terrestrial snail guts also contain a majority of Enterobacteriaceae members, including the genera *Butiauxella*, *Citrobacter*, *Enterobacter*, and *Kluyvera* [62]. One species from genus *Sphingobacterium*, *S. multivorum*, has been isolated from the giant African land snail *Lissachatina fulica* [23, 63]. The higher proportion of Proteobacteria could be attributed to any number of life history traits, such as diet, trophic level, farmed versus wild species, method of reproduction, and infection with parasites.

Members of the gut microbiome are often expected to aid in food digestion and nutrient absorption of the host [9]. In snails, food is scraped by the radula and combined with salivary gland secretions and digested in the stomach [23]. In this process, gut bacteria play an important role in capturing energy from digested plant biomass. Snails may use their resident gut bacteria to degrade and ferment cellulose, hemicellulose, and lignin, all of which are common to many of their diets, though such functions were not deduced in this data set [23].

Overall, the known symbionts associated with the *O. strigosa* gut are expected to aid digestive functioning consistent with the inferred diet of this snail. As with some other land snails, *O. strigosa* feeds preferentially on decaying wood and leaf litter rather than fresh leafy greens [28]. As such, bacterial symbionts with functional ability to help the host digest complex molecules, like lignocellulosic matter, are consistent with the needs of this snail host. Cellulose degrading bacteria isolated from the gut of different snails are also found in *O. strigosa*, these include many members of *Enterobacter*, *Bacillus*, and members of genus *Sphingobacterium*, including *S. multivorum*. Lactic acid bacteria, responsible for fermentation in other snail guts, were similarly also found in our samples, including members of *Enterobacter*, *Lactococcus*, *Butiauxella*, and *Enterococcus* (like *E. casseliflavus*) [23]. Although we cannot test bacterial functions directly with our microbiome profiling data, it is likely that *O. strigosa* gut microbiome aids its digestive and fermentative processes.

#### Core gut microbiome

Venn analyses found that 11.34% (800 OTUs) were common to all three treatment groups of the total 7056 OTUs identified (Fig. 2B). The non-starved adult snails share 22.15 and 49.42% OTUs with the fetal snails and starved snails respectively.

To further determine the members of the core microbiome, which is the most stable part of the microbiome, we first identified the families and genera that were present in all treatment groups of individuals. Of the 800 common OTUs, the largest proportion of OTUs came from families Sphingomonadaceae, Chitinophagaceae, Flavobacteriaceae, and Sphingobaceteriaceae, and within those, genera *Sphingomonas*, *Flavobacterium*, and *Pedobacter.* The most common genus found across all groups was *Sphingomonas* (4.34% of the common OTUs).

We then determined which OTUs were present in 100% of samples. Only four OTUs were common to every sample’s gut microbiome: OTU_9 and OTU_10, unidentified members of family Enterobacteriaceae; OTU_1, *Sphingobacterium faecium*; and OTU_17, an unidentified member of genus *Sphingomonas*. Together, these four OTUs contributed to roughly 50% of total reads. 19 OTUs were found in at least 90% of all snail samples, which made up roughly 62% of all bacterial reads. The 43 OTUs found in at least 80% of all snail samples made up 68% of all bacterial reads.

The fact that the four core gut microbes contribute to half of the relative abundance across all samples might reflect their ecological importance as beneficial symbionts to the snail host. It is worthy to note that these bacterial strains were found in the unborn fetal snails, suggesting that they may be passed down through vertical transmission (see discussion in fetal and adult comparison section) from parent to offspring. Therefore, these strains may confer some evolutionary advantage at birth for these snails. However, it is important to note that functional capacity of such microbes was not examined in this study. Members of Enterobacteriaceae and Sphingobacteriaceae are known cellulose-degrading bacteria in other snail species [23]. While less functional information is known regarding *Sphingomonas* species, members of the genus have been found in the freshwater snail *Biomphalaria glabrata* and are thought to play a role in immune functioning, specifically in parasite defense [64]. In the land snail *Cornu aspersum,* cellulose-degrading bacteria and lactic acid bacteria persisted in every life stage of the snail examined, which is consistent with the four core bacteria found here [65]. This demonstrates that *O. strigosa* likely maintains an obligate group of bacterial symbionts that are important to the snails’ survival.

### Comparison between treatment groups

#### Taxonomic composition

##### Fetal vs. adults

Fetal gut microbiome samples consisted of fewer OTUs than their adult counterparts. In fetal samples, a total of three phyla accounted for 97.32% of the total sequences. Proteobacteria (86.92%) was the most dominant phylum followed by Bacteroidetes (8.03%) and Firmicutes (2.37%). The lack of Verrucomicrobia at abundances > 1% in fetal samples, as opposed to the phylum’s larger presence in every other group, indicates taxa from this phylum might be horizontally acquired in adults. Some snails are known to eat soil to augment their gut microbiome [23]. Verrucomicrobia is generally abundant in soil and freshwater samples, and so could likely be found in the soil substrate that *O. strigosa* live and burrow in. Other phyla that were common in similar abundances across snail groups, like Proteobacteria, Bacteroidetes, and Firmicutes, may be vertically transmitted from parent to offspring. A small number of OTUs were present only in fetal samples and not the other treatment groups, this indicates that vertical transmission of highly transient bacteria may exist, explaining why these OTUs were not found in adults.

The clustering of gut bacteria by host life stage was highly significant in the PERMANOVA analysis. A non-metric multi-dimensional dimensional scaling (NMDS) plot showed that fetal samples formed a distinct cluster but could not be separated from adult snail samples (Fig. 2C, upper). The R^2^-value indicates that the variability attributed to life stage as a factor is about 9.5% (PERMANOVA: *p*-value < 0.001, *R*^*2*^-value = 0.095).

Fetal *O. strigosa* are an important group for studying how life stage influences gut microbiota because unlike many other land snails, these snails are ovoviviparous and give live birth. Rather than extracting DNA from an egg mass, we can dissect the fetal snails directly from the parent to reduce the impacts of any environmental contamination. In other animals, fetal microbiomes are similarly present, whether acquired directly from the mother’s oviduct or through environmental seepage, like through eggshell pores [17]. The majority of existing literature looking at microbiome changes over varying life stages use vertebrate species as models. Therefore, much of the following discussion compares our study to these vertebrates. For example, chickens show a similar pattern of higher relative abundance of Proteobacteria earlier in life. Members of Proteobacteria in chicks and in snails may be poor competitors that are unable to compete and persist in a mature gut microbiome [4]. As young chickens advance in age, even beginning as early as seven days old through 42 days, their microbiome becomes more diverse, showing increased relative abundances from more phyla [17]. This is because gut microbiome succession is dependent on nutrition and the establishment of new bacteria through exogenous food sources [4]. As a case in point, when rabbits are fed only milk in the first days of life, they possess no cellulolytic bacteria and are unable to digest plant matter [66]. Since some bacteria is found in unborn, fetal snails, they may have an advantage in already possessing some mutualistic gut bacteria. As snails age and ingest food, the gut microbiome likely becomes more and more diverse until it reaches the composition of the adult microbiome. Further studies of varying life stages of *Oreohelix* could provide more detailed insight into how changes in diet and age can affect the makeup of the gut microbiome.

Means of reproduction may also influence microbiome composition. Asexually reproducing New Zealand mud snails (*Potamopyrgus antipodarum)* are dominated by a strain from the genus *Rhodobacter*, while sexually reproducing individuals are dominated by a strain from the order Rickettsiales. This association suggests the snail’s reproductive strategy has certain levels of influence on the assembly of its microbiota [67]. *Oreohelix strigosa* can self-fertilize or mate, but seem to prefer mating as little research has investigated self-fertilization ([68], personal observation). Means of reproduction may be an important factor that alters the microbiome of fetal snails in *Oreohelix*, but this needs further investigation.

##### Starved vs. non-starved

Starved snails had ten phyla showing relative abundances greater than 1%, compared with five in the adult group and three in the fetal group. Starved snails showed increased relative abundances of the phyla Acidobacteria, Planctomycetes, Cyanobacteria, Spirochaetes, and Tenericutes, which are present in the other two treatment groups but at low abundances. As this is proportional data, this trend can be also caused by originally abundant taxa diminishing, resulting in the appearance of increasing abundances of other phyla.

A non-metric multidimensional scaling (NMDS) plot showed that adult starved gut microbial compositions were not distinctively separate from adult non-starved samples. Visually, the starved samples clustered within the greater cluster of non-starved samples (Fig. 2C, lower). PERMANOVA analyses showed the effect of starvation, though significant, explained only 3.08% of the variance between samples (PERMANOVA: *p*-value < 0.002, *R*^*2*^-value = 0.038).

Starved sample microbiomes appear to be largely a subset of adult sample microbiomes. Only 119 OTUs were unique to starved samples, compared with over 2000 in adults. Other studies have found decreased OTU diversity in starved organisms. The gut of tunicate *Ciona intestinales* shows a similar pattern of OTU presence in starved and non-starved individuals; a relatively small amount of OTUs was shared between these two groups [69]. *C. intestinales* also similarly showed a greater number of unique OTUs in only the non-starved group, compared with a smaller number (still greater than the number of OTUs found in both groups) found only in the starved group [69]. This trend is also shown in fresh-water crayfish, where starved samples showed genera (largely from genus *Vibrio*) that were present, but rarer in fed samples in high relative abundances [70]. Unlike our study, these genera came to make up the majority of the starved microbiome, rather than simply becoming more abundant [70]. This discrepancy may be due to the much longer starvation period in the crayfish study that may have allowed *Vibrio* species the time needed to replace core bacterial species (four weeks, compared with our one week period). Prolonged starvation periods may give the microbiome more time for rarer species to take over the community, giving way to a rapid shift in bacterial diversity and relative abundance of some species. However, it is likewise difficult to denote a universal response against starvation, as tolerance levels vary across different host species [70, 71]. Future studies should attempt to increase the length of the starvation period and use a time series analysis to see if similar trends occur in *Oreohelix*.

Lastly, we conducted the same PERMANOVA test using only samples found in one location that included both starved and fetal groups (MRS), to validate the results without any possible impact of locality. In this test, the significant results of age and starvation hold up, with age explaining roughly 9.3% of the variation between samples (*p*-value < 0.001) and starvation explaining 5.5% (*p*-value < 0.05).

#### Diversity metrics

We used the non-parametric Kruskal-Wallis test to test for significant differences among the means of the richness, evenness, and Shannon index of the three groups (non-starved, starved, and fetal). The tests returned significant *p*-values for each metric (Evenness: Kruskal-Wallis chi-squared = 10.464, df = 2, p-value = 0.005342; Richness: Kruskal-Wallis chi-squared = 13.233, df = 2, p-value = 0.001338; Shannon Index: Kruskal-Wallis chi-squared = 13.156, df = 2, p-value = 0.001391, Fig. 2D). The starved group showed significant higher evenness compared to others, and the fetal group showed significant lower mean richness and Shannon diversity. In the bootstrap analyses where we compared fetal and starved samples with random samples of 6 and 12 non-starved adults, the significantly higher microbiome evenness in starved snails was found in 48% of the bootstrapped replicates; the lower Shannon Index scores in fetal samples was true for 52% of the replicates; and the lower fetal microbiome richness was supported by 93% of the replicates.

##### Fetal vs. adult

Microbial ecology theory predicts that microbial species passed vertically from parent to offspring are more likely to be beneficial to the host, while species gained horizontally from the environment are opportunistic and more variable [72]. Fetal snails unexposed to their surrounding environment, are also unlikely to gain symbionts horizontally. This is consistent with our finding of a significant lower richness in fetal snails compared with adult snails.

Across both vertebrates and invertebrates, there are many examples of vertical transmission of microbiomes. Demonstrated examples include insects [73, 74], sponges [75], bivalves [76–78], and cephalopods [79–82]. In sponges, members of up to ten bacterial phyla and one archael phylum have been vertically transmitted from adult sponge to other life stages, including oocytes of oviparous sponges and embryos of viviparous sponges [83]. In chemosynthetic vesicomyid clams, vertical transmission also appears to be the main mechanism for maintaining thioautotrophic bacterial symbionts [76, 83]. In many of these animals, the deposited microbiome is not as rich as the adult microbiome, and compositional changes happen throughout maturation likely due to further horizontol transmission. For example, amylolytic bacteria are vertically transmitted in the land snail *Cornu aspersum*, whereas transient proteolytic and cellulolytic bacteria are gained through environmental augmentation when the adult snail is active [23, 65]. Importantly, the fetal snails examined here were unborn and therefore completely unexposed to their surrounding environment, so examining more life stages of these snails, like juveniles, could shed more light on which bacteria are adopted from parents versus environments.

##### Starved vs. non-starved snails

Starved samples showed significantly greater evenness than non-starved adults, but not always in the bootstrap analyses. Starved samples also showed nonsignificant differences to richness and Shannon Index scores compared with adults.

Limited studies have assessed the effect of starvation on the gut microbiome in animals. The gut microbiome in cod [84], seabass [85], and shrimp [86] indicate that they can rapidly adapt to starvation stress. In shrimp, diversity indices between starved and non-starved groups were similarly not significantly different from one another. This lack of overall difference in community structure is hypothesized to be attributed to a few microbial members being more sensitive to disturbance, while the greater community is resistant [87]. These few members could be microbial “gatekeepers,” which contribute disproportionately to the functioning of the gut microbiome and the overall health of the host. If these gatekeepers are lost due to starvation stress, profound shifts in function of the gut microbiome could occur [86]. So, while diversity measures like richness between starved and non-starved groups are non-significantly different in this study, there may be significant changes happening in the actual functioning of the gut microbiome. Future studies should utilize -omics methods (e.g. metagenomics) to investigate how functional roles of the microbiome change due to starvation stress.

In addition, the lack of significant responses of richness and diversity, compared with higher evenness may be because of dysbiosis in the snail. Particularly, this may be consistent with the “Anna-Karenina Principle” for bacterial microbiomes in response to stress [88]. Following this principle, a host may not be able to regulate its microbiome when experiencing stress, and thus its microbial community takes on a more randomly distributed community structure. Thus, host individuals will not show consistent microbial community shifts in response to disturbance, though an overall separation between stressed and non-stressed populations may be obvious [89]. In invertebrates, stressed coral populations have also been reported to show increased dispersion in their microbial communities [88]. Similarly, ocean acidification can increase microbiome variability in sea sponges [90]. The effects of dysbiosis in starved organisms are important to elucidate as a transformed gut microbiome can lead to altered, poorer immune functioning in hosts and increase risk of disease from pathogenic bacteria [70, 91]. Signs of dysbiosis in snails may be particularly useful for researchers of montane ecosystems because snails act as bioindicators – their highly sensitive disposition reflects the impact of climatic stress on their environment [92].

There does not seem to be consistent microbiome responses to starvation across different animal groups. For example, in contrast to our results, the evenness of starved abalone was lower than fed abalone [93]. Due to a wide variety of microbiome responses across animals, the effect of starvation may need to be examined on a species-by-species basis. As with other studies [94], it may be useful to conduct a starvation study across a diverse array of gastropods to ascertain any shared responses.

Another reason why a universal response to starvation cannot be detected is because many host species go through fasting periods in their natural life cycle. For example, crustaceans must fast while they molt [95]. The Chinese alligator [96] is a natural hibernator that can fast for several months due to lower temperatures and unavailability, as are many mammals including bears [97] and squirrels [98]. In all of these species, microbial diversity has been impacted due to seasonal fasting, though not necessarily in a negative way. *Oreohelix strigosa* typically enters a dormant state of aestivation during periods of hotness, dryness, or freezing temperatures that would lead to desiccation [99]. Therefore, there are many times when feeding is not a daily occurrence. The unpredictability of *O. strigosa’s* lifestyle may enable more gut microbiome adaptability to periods of fasting, therefore not showing dramatic changes in diversity patterns.

Lastly, we must acknowledge that the small sample sizes of our fetal and starved groups limit the statistical power of our experiment. We have used a sensitivity analysis to best assess whether our results stand with more balanced groups. However, to further elucidate the effects of life stage and starvation on gut microbiome composition and diversity, we recommend that follow-up studies integrate a higher amount of replication in their investigations.

## Conclusion

Recent studies have reported the importance of gut microbiome to the health of hosts across the animal kingdom [100, 101]. Hence, the main purposes of this study were to characterize the bacterial diversity in both adult and fetal *Oreohelix strigosa*, and to determine the diversity and composition of bacterial communities in starved and non-starved snails.

This study has led to several major findings, including that *O. strigosa* showed a rich and diverse microbiome consisting largely of members of Proteobacteria, more specifically, Gammaproteobacteria. This gut microbiome is more diverse than that of many other gastropod species. We hypothesize that the function of this gut microbiome is to aid in digestion, specifically helping to digest tough, cellulolytic matter typical of this snail’s diet. A core gut microbiome was found in members of *O. strigosa*. Specifically, we were able to determine that four OTUs were found across the two life stages of snails studied and all localities. The presence of select bacterial species regardless of environmental variables, such as geographic location, indicates that these bacteria are likely invaluable to *O. strigosa’s* survival. The presence of a gut microbiome particularly in fetal snails lends credence to the hypothesis that some bacterial members are vertically transmitted from parent to offspring before birth. More extensive studies looking at a wider range of variables are needed to confirm such patterns. Overall, the gut microbiome of *O. strigosa* is a diverse community, with great potential for increased research in both its natural habitat and manipulated lab studies.

## Supplementary Information

**Additional file 1.**

## Data Availability

The sequencing dataset supporting the conclusions of this article is available in the FigShare repository, 10.6084/m9.figshare.14558502.v1 and 10.6084/m9.figshare.14558496.v1.

## Abbreviations

NMDS: Non-metric multi-dimensional scaling
OTU: Operational taxonomic unit
PERMANOVA: Permutational analysis of variance
